# p65/miR‐23a/CCL22 axis regulated regulatory T cells recruitment in hepatitis B virus positive hepatocellular carcinoma

**DOI:** 10.1002/cam4.2611

**Published:** 2019-11-25

**Authors:** Zhi‐Qin Li, Hong‐Yan Wang, Qing‐Lei Zeng, Jing‐Ya Yan, Yu‐Shu Hu, Hua Li, Zu‐Jiang Yu

**Affiliations:** ^1^ Department of Infectious Disease The First Affiliated Hospital of Zhengzhou University Zhengzhou Henan P.R. China

**Keywords:** CCL22, HBV, hepatocellular carcinoma, MiR‐23a, p65, Tregs recruitment

## Abstract

**Background:**

CCL22 played critical roles in Tregs recruitment. The upstream regulators modulating CCL22 in hepatocellular carcinoma (HCC) were not clearly understood.

**Methods:**

MiR‐23a, p‐p65, p65, CCL22, and Foxp3 levels were monitored by RT‐qPCR and western blotting. Immunofluorescence assay was used to perform the costaining of Foxp3 and CD4 on liver tissues. Transwell assay was applied to evaluate the migration ability of Tregs. Dual‐luciferase assay was performed to determine relationship of miR‐23a/CCL22 and p65/miR‐23a. Chromatin immunoprecipitation (ChIP) was applied to detect the direct binding of p65 to miR‐23a promoter. Xenograft tumor models were developed to investigate the functions of p65 and miR‐23a in vivo.

**Results:**

HBV infection was associated with reduced survival and increased Tregs recruitment in HCC patients. MiR‐23a was decreased, whereas p65, CCL22, and Foxp3 were increased in HBV^+^ tumors. MiR‐23a was inversely correlated with CCL22 and Foxp3 expression in HCC. MiR‐23a directly targeted CCL22 3’UTR, leading to CCL22 reduction and attenuated Tregs recruitment. Meanwhile, p65 functioned as a transcription repressor of miR‐23a by directly binding to its promoter. Inhibition of p65 induced miR‐23 expression, leading to less CCL22 expression and Tregs recruitment in vitro. CCL22 was the indispensable effector underlying p65/miR‐23a axis and Tregs recruitment. MiR‐23a inhibitor promoted xenografted tumor growth accompanying with upregulation of CCL22, whereas p65 inhibition exerted opposite effects.

**Conclusion:**

Blockage of p65 disinhibited miR‐23a expression, leading to CCL22 reduction and repress Tregs recruitment. Targeting p65/miR‐23a/CCL22 axis was a novel approach for HBV^+^ HCC treatment.

## Graphical abstract

MiR‐23a expression is significantly decreased in HBV+ hepatocellular carcinoma tissues. MiR‐23a was inversely correlated with CCL22 and Foxp3 in HBV+ hepatocellular carcinoma tissues. MiR‐23a directly targeted CCL22 to attenuate Tregs recruitment.

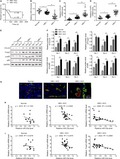

## 1 INTRODUCTION

Liver cancer is the fourth most common cancer in China. It was estimated that around 466 thousands of newly diagnosed liver cancer would be reported and caused 422 thousands of death every year in China.^1^ On the other hand, it was found that around 80% of liver cancer patients were hepatitis B virus (HBV) carriers in China.^2^ This indicates that HBV is a critical risk factor for liver cancer development. Given that a large number of population are infected with HBV in China, researches that aim to decipher HBV‐mediated liver cancer development and progression are extremely important.

Increasing evidences demonstrated that immune evasion of tumor cells contributed to cancer progression.^3^ Regulatory T cells (Tregs) are a subpopulation of T cells with immunosuppressive capability and have been frequently identified to play critical roles in immune evasion of cancers.^4^, ^5^ A meta‐analysis using data from 1279 hepatocellular carcinoma (HCC) patients and 547 healthy controls revealed that the ratio of circulating and tumor‐infiltrating Tregs was significantly higher in HCC patients than healthy controls.^6^ Meanwhile, high level of Tregs in hepatocellular carcinoma microenvironment represented a poor prognosis with short overall and disease‐free survival.^7^ More importantly, depletion of Tregs by antibodies or targeting signaling in Tregs was reported to be effective treatments for tumor growth and metastases of hepatocellular carcinoma in the preclinical models.^8^, ^9^ Recently, cancer immunotherapies which target immune checkpoints and recover immune response in tumors were showing great successes in several clinical trials and appeared to be the most promising cancer treatments in future.^10^, ^11^ Based on these findings, targeting the key pathways or molecules that drive intratumoral recruitment of Tregs may hold major potential for clinical application.

C‐C Motif Chemokine Ligand 22 (CCL22) is a potent chemoattractant for T‐lymphocytes, including Tregs.^12^, ^13^ It was known that tumor cells‐derived CCL22 functioned as a key factor for intratumoral recruitment of Tregs in several types of cancers, including liver cancer.^8^, ^14^ Blocking of CCL22 therefore may represent a novel approach for suppression of Tregs recruitment and subsequent tumor growth.^15^ Interestingly, bioinformatics analysis predicted that 3’UTR of CCL22 contained a putative‐binding site of miR‐23a seed sequence. It could be expected that miR‐23a‐mediated CCL22 degradation might lead to the attenuation of Tregs recruitment into tumor tissues. Interestingly, the tumor suppressive functions of miR‐23a have been reported in several types of cancers, including liver cancer.^16^, ^17^ For example, it was reported that berberine‐mediated antiproliferation and proapoptosis effects required miR‐23a expression in HCC cells.^16^ In this respect, investigation of miR‐23a and CCL22 relationship may provide a novel strategy for anticancer therapy, yet it has not been studied in HCC.

In order to enhance the feasibility of miR‐23a targeting in clinical practice, identification of upstream modulator of miR‐23a will be of great interest to researchers. Bioinformatic analysis revealed that there was at least one p65‐binding site in the miR‐23a promoter, suggesting that p65 could be a modulator of miR‐23a expression.

In this study, we hypothesized that there was a p65/miR‐23a/CCL22 axis driving Tregs recruitment in HCC. Inhibition of p65 might induce the expression of miR‐23a, which then reduces CCL22 level and attenuates Tregs recruitment in HCC. Given that HBV infection is featured on HCC, the comparisons of miR‐23a, p65, and CCL22 expression between HBV^‐^ and HBV^+^ patients or cell lines were also performed. To our best knowledge, this is the first work that focused on the mechanism of p65/miR‐23a/CCL22 axis‐mediated Tregs recruitment in HCC.

## 2 MATERIALS AND METHODS

### 2.1 Human samples

Human tissues were collected and used under a protocol approved by the Ethics Committee of the First Affiliated Hospital of Zhengzhou University (2018‐KY‐60). The informed consent were signed by all patients. Adjacent normal liver tissues were obtained from 15 patients. Hepatocellular carcinoma (HCC) tissues were obtained from 30 HBV infection negative (HBV^−^) and 30 HBV infection positive (HBV^+^) cancer patients. For the survival analysis, the long‐term follow‐up data of HBV^+^ HCC patients (n = 30) and HBV^‐^ HCC patients (n = 30) were collected from the First Affiliated Hospital of Zhengzhou University. The tissue samples were immediately frozen in liquid nitrogen till further assays. The clinicopathological features of all HCC patients were shown in Table 1.

**Table 1:** The clinicopathological characteristics of hepatocellular carcinoma patients

Clinical parameters	HCC		*P* value
	HBV‐negative (n = 30)	HBV‐positive (n = 30)	
Gender			
Male	13	15	.7961
Female	17	15	
Age			
<60	12	17	.3015
≥60	18	13	
TNM stage			
Ⅰ + Ⅱ	18	10	.0692
Ⅲ + Ⅳ	12	20	
Tumor diameter			
≤50 mm	19	14	.2993
>50 mm	11	16	
Distant metastasis			
Yes	9	19	.0191
No	21	11	

### 2.2 Cell culture and treatment

The human embryonic hepatocytes cell line WRL68, hepatocellular carcinoma cell line HepG2 (HBV^−^) and human embryonic kidney cell line HEK293 were obtained from American Type Culture Collection (ATCC). HepG2.2.15 cells are stablely HBV‐transfected cells derived from a hepatoblastoma HepG2 cell line (Chongqing Medical University). All the cells were maintained in Dulbeco's Modified Eagle Medium (DMEM, Gibco) supplemented with 10% of heat‐inactivated fetal bovine serum (Gibco) and 1% of Penicillin‐Streptomyicin solution (Gibco) in a humidified atmosphere of 5% CO_2_ at 37°C. In some experiments, HepG2 or HepG2.2.15 cells were treated with 5 μmol/L parthenolide (a p65 inhibitor, TOCRIS) or miR‐23a mimics (GenePharma) for 24 hours. For rescued study, cells were pretreated with miR‐23a inhibitor (GenePharma) following by 5 μmol/L parthenolide for 24 hours.

### 2.3 Immunofluorescence assay

Sections were sliced at a thickness of 5 μm from paraffin‐embedded tissues blocks, following by deparaffinization and rehydration under a standard protocol. After antigen retrieval with citrate buffer solution (pH 6.0), the sections were treated with 3% hydrogen peroxide in methanol, blocked with 5% goat serum in PBS‐T (0.3% Triton X‐100 in PBS) for 30 minutes, and incubated with primary antibodies anti‐Foxp3 (PA1‐46126, ThermoFisher Scientific) and anti‐CD4 (MA1‐10800, ThermoFisher Scientific) for overnight at 4°C. Fluorescent dye‐conjugated secondary antibodies (ThermoFisher Scientific) were then applied to label and visualize the expression of target proteins in tissues.

### 2.4 Dual‐luciferase reporter assay

The reporter assays were performed to investigate the modulation of miR‐23a promoter by p65 and CCL22 3′UTR by miR‐23a. For promoter assay, miR‐23a promoter containing predictive p65‐binding site or corresponding mutant promoter was cloned into pGL3‐basic vectors. pcDNA3.1‐p65 was applied for p65 overexpression. pRL Renilla Luciferase vectors were used as the control plasmid. During transfection process, pGL3:pcDNA3.1‐p65:pRL (25:25:1) were cotransfected into HEK293 cells with lipofectamine 3000 (ThermoFisher Scientific) at a ratio of 1:3 (DNA: lipofectamine). As a control, pcDNA3.1 vectors were also applied for cotransfection in the separated experiments as above. After transfection for 48 hours, firefly luciferase activities were determined and adjusted by Renilla luminescence using the kit according to the manufacture's instruction (Promega).

For 3′UTR assay, oligos containing putative miR‐23a‐binding site were cloned from CCL22 3′UTR and inserted into pmirGLO vectors (Promega). The mutant was generated using the Phusion site‐directed mutagenesis kit (F541, ThermoFisher Scientific) according to manufacturer's instruction. Briefly, phosphorylated mutagenic primers consisting of mismatch base were designed and applied for linear plasmid amplification using Phusion polymerase. The linear plasmid was then recirculated by T4 ligase and transformed for subsequent manipulation. MiR‐23a mimics or scramble control were obtained from GenePharma. pRL vectors were used as the transfection control. During transfection process, miR‐23a mimics and pmirGLO vectors were cotransfected into HEK293 cells with lipofectamine 3000 at a ratio of 1:3 (DNA: lipofectamine). After transfection, the luminescence among groups were detected as above.

### 2.5 Chromatin immunoprecipitation (ChIP) assay

ChIP assay was performed using the ChIP‐IT Express Enzymatic kit (Active Motif). The protocol was adopted according to manufacturer's instruction. Anti‐p65 (#8242, 1:100) and its isotype IgG control (#3900, 1:100) were obtained from Cell signaling technology. HEK293 cells were cross‐linked with formaldehyde, following by enzymatic shearing and antibody incubation for overnight at 4℃. Protein‐DNA complex was then immunoprecipitated with protein G magnetic beads. The p65‐binding chromatin was eluted, reversed for cross‐linking, digested with proteinase K and then purified with the DNA columns. The DNA was then assessed by quantitative PCR.

### 2.6 Cell sorting

Treg cells were purified from healthy donors using Human Regulatory T cell Sorting Kit according to manufacturer's instructions (BD Pharmingen). Peripheral blood mononuclear cells (PBMC) were isolated from 30 mL whole blood with Ficoll‐Paque PREMIUM density gradient media (GE Healthcare Bio‐Sciences). This was followed with antibody cocktail staining and flow cytometry sorting. The identification of Treg cells was determined by Foxp3 and CD4/CD25 costaining with flow cytometry. The fraction of CD4^+^/CD25^+^ were collected for subsequent cell migration test.

### 2.7 Transwell migration assay

The 24‐well Transwell culture inserts with 5‐μm pore size were applied. Supernatant of HepG2 or HepG2.2.5 cells which were treated with p65 inhibitor/vehicle control or miR‐23a mimics/inhibitor/scramble control was seeded onto the lower chamber with 400 μL of culture medium. For p65 inhibitor study, parthenolide, a p65‐specific inhibitor, or its vehicle control (DMSO) was treated cells 24 hours before Treg coincubation. For microRNA study, cells were transfected with miR‐23a mimics/inhibitor or scramble control 24 hours before coincubation. Purified Treg cells (1 × 10^5^/well) were added to the upper chamber of the transwell and coincubated with conditioned medium for 24 hours at 37°C. For neutralization assay, anti‐CCL22 antibody (0.5 μg/mL) or isotype IgG control was also added into the conditioned medium. The migrated Treg cells were counted by researchers who were blinded to the experimental design.

### 2.8 Stable cell line establishment

pGCMV/EGFP/miR/Blasticidin plasmids containing miR‐23a inhibitor sequence or its scramble control were ordered from GenePharma. The plasmids were transfected into HepG2.2.15 cells with Lipofectamine 3000 in a ratio of 1:3 (DNA:Lipofectamine). Monoclonal cell lines that stably express miR‐23a inhibitor (HepG2.2.15/miR‐23a‐inhibitor) or scramble control (HepG2.2.15/NC) were then selected with Blasticidin (10 µg/mL, TOCRIS) and then used to nude mice xenograft study.

### 2.9 Xenograft tumor model

Six‐week‐old male BALB/c nude (nu/nu) mice were purchased from Shanghai SLAC Laboratory Animal Center. The nude mice xenograft study was approved by the Animal Ethics Committee in the First Affiliated Hospital of Zhengzhou University. HepG2.2.15 cells which stably express miR‐23a inhibitor (HepG2.2.15/miR‐23a‐inhibitor) or scramble control (HepG2.2.15/NC) were subcutaneously inoculated in the nude mice. In the groups containing p65 inhibitor treatment, 5 mg/kg parthenolide was applied via tail intravenous injection. The xenograft tumor size was measured with a vernier scale for every 5 days. After 30 days, mice were euthanized by cervical dislocation. The tumor tissues were harvested for volume and weight measurement, protein isolation and western blotting analysis.

### 2.10 RNA isolation and quantitative PCR (qPCR)

Total RNA from human tissues or cell lines was extracted with Trizol reagent (ThermoFisher Scientific) according to the manufacturer's instruction. Complementary DNA (cDNA) was synthesized from 1 μg of total RNA using the PrimeScript RT reagent Kit (Takara). qPCR was performed with SYBR Premix EX Taq^TM^ kit (Takara) in an ABI 7500HT real‐time PCR system (Applied Biosystems). GAPDH and U6snRNA were used as an internal control for mRNA and miRNA, respectively. Relative expression levels were calculated by the 2^−ΔΔCq^ method.

### 2.11 Western blotting

Primary antibodies against Foxp3 (#12632, 1:1000), p‐p65 (#3033, 1:1000), p65 (#8242,1:2000), GAPDH (#5174, 1:1000) and secondary antibodies (#7074, 1:3000) were obtained from Cell signaling technology. Anti‐CCL22 antibody (MAB336, 1:1000) was ordered from R&D systems. Total protein was extracted with cell lysis buffer (50 mmol/L Tris, 150 mmol/L NaCl, 1% NP‐40, 1 mmol/L EDTA, pH 7.6) containing a cocktail of protease inhibitors. Protein concentration was determined using Pierce BCA protein assay kit (San Jose, CA, US) according to manufacturer's instruction. 30 µg protein samples were separated on SDS‐PAGE gels, then transferred onto PVDF membranes (0.22 μm pore, Roche). After blocking with TBST buffer (20 mmol/L Tris, 137 mmol/L NaCl, 0.1% Tween‐20, pH 8.0) containing 5% nonfat milk, membranes were incubated with primary antibodies overnight at 4°C. Then membranes were incubated with secondary antibody for 1 hour at room temperature. The protein bands were visualized using Immobilon Western Chemiluminescent HRP substrate (Millipore, Burlington, MA, US). The proteins were quantified using Quantity One software (Bio‐Rad Laboratories, Inc).

### 2.12 Statistical analysis

All the experiments were performed for three times, data were expressed as mean ± standard deviation (SD). Statistical analyses were performed by GraphPad Prism 6 (GraphPad Software, Inc). Unpaired two‐tailed *t* test was used for comparison between two groups. One‐way analysis of variance (ANOVA) followed by Tukey post hoc test was used for multiple comparison. The Kaplan‐Meier estimate was applied to compare the overall survival time between HBV^+^ and HBV^−^ cancer patients. The correlation between HBV^+^ and HBV^−^ cancer patients and clinicopathological characteristics of patients was assessed by the Chi‐squared test. The significance of difference was determined as indicated in the figure legends. *P < .05 was considered significant.

## 3 RESULTS

### 3.1 Lower miR‐23a expression was correlated with higher level of CCL22 expression and intratumoral Treg recruitment in HBV‐positive HCC

**Figure 1:**
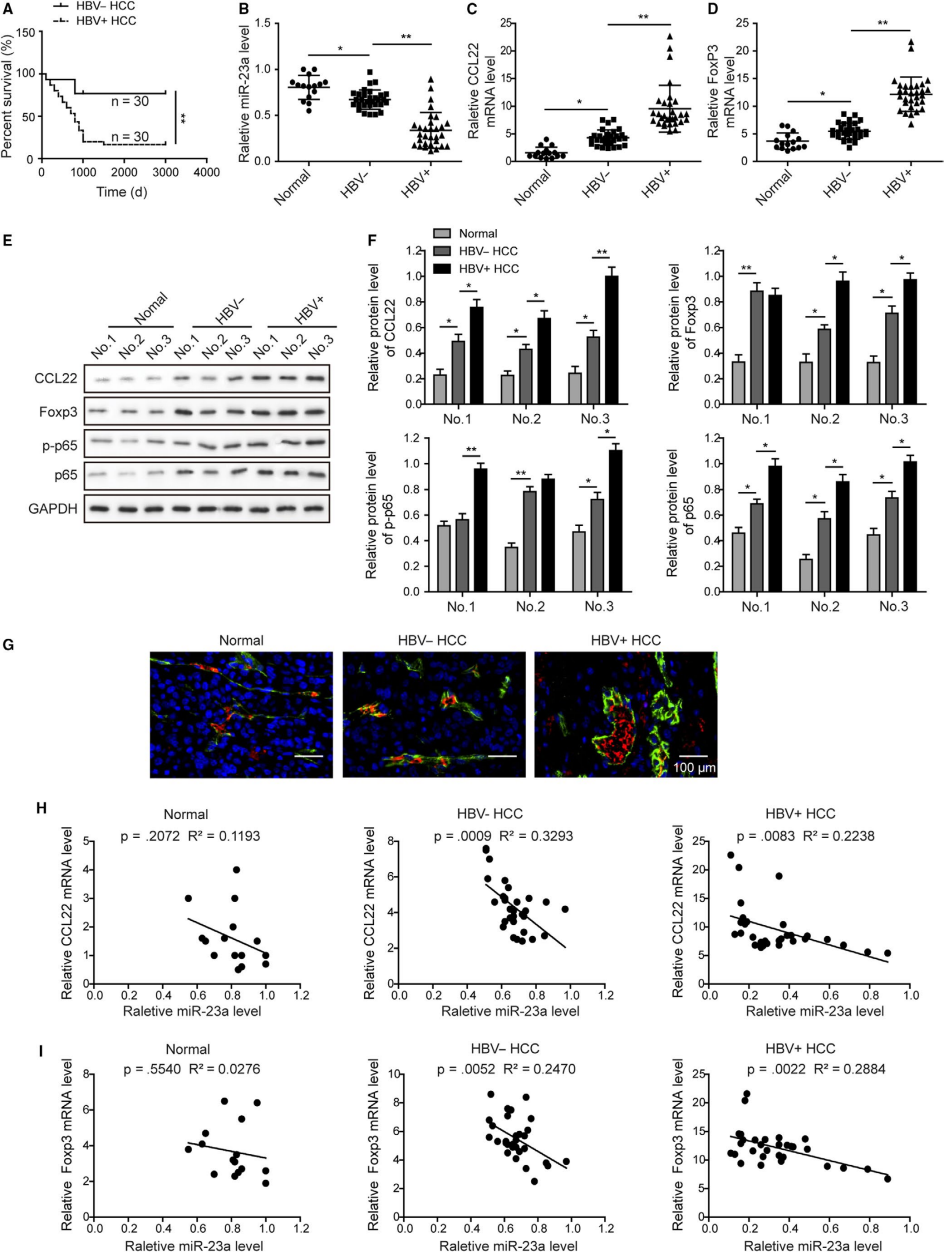
Lower miR‐23a expression was correlated with higher level of CCL22 expression and intratumoral Treg recruitment in HBV‐positive HCC. A, HBV infection was associated with patient survival rate of HCC. Patients carrying HBV (n = 30) had significantly poorer prognosis than HBV^‐^ patients (n = 30). B, HBV^‐^ tissues (n = 30) and HBV^+^ tissues (n = 30) expressed significantly lower level of miR‐23a. C, HBV^−^ tissues and HBV^+^ tissues expressed significantly higher mRNA level of CCL22. D, HBV^‐^ tissues and HBV^+^ tissues expressed significantly higher mRNA level of Foxp3. E, The protein levels of CCL22, Foxp3, p‐p65, and p65 in normal control, HBV^‐^ and HBV^+^ tumor tissues were evaluated by western blotting. F, The gray scale analysis of CCL22, Foxp3, p‐p65, and p65 in normal control, HBV− and HBV+ tumor tissues. In ascending order: normal < HBV^−^ < HBV^+^. G, Foxp3 signals (red) were colocalized with CD4 (green) signals in tissues. The ratios of Foxp3^+^CD4^+^ cells were gradually increased from normal, HBV^−^ HCC to HBV^+^ HCC tissues. H. MiR‐23a level was inversely correlated with CCL22 expression in HCC tissues, but not in normal control. I. MiR‐23a level was inversely correlated with Foxp3 expression in HCC tissues, but not in normal control. Error bars represented mean ± SD. **P < .01 and *P < .05. HBV^+^, HCC tissues with HBV infection. HBV^−^, HCC tissues without HBV infection. Normal, normal liver samples

To investigate the roles of potential Tregs modulators, we explored whether the expression levels of miR‐23a, CCL22, and Foxp3 were associated with the progression of patients. First, the long‐term follow‐up was conducted for up to 3000 days after hospital discharge. As presented in the Figure 1A, HBV infection significantly reduced the overall survival time of cancer patients than noncarriers. As shown in Table 1, HBV infection was associated with distant metastasis (*P* < .05). This indicated that HBV could be a critical factor of tumor progression. To explore the mechanism of HBV‐related tumor development, miR‐23a was investigated in this study. Consistent with previous reports in other tumors, it was found that miR‐23a level was significantly lower in both types of tumors (HBV^−^ and HBV^+^) than in normal controls. Notably, significant difference in miR‐23a was also observed between HBV^+^ and HBV^−^ tumors, HBV infection appeared to further reduce the expression of miR‐23a (Figure 1B). This observation indicated that miR‐23a might play a role in HBV‐mediated tumor progression. Of note, CCL22, a key gene mediates Tregs recruitment and tumor immune evasion, was found in the candidate list. As expected, both CCL22 and Foxp3 (marker of Tregs) mRNA levels were increased in tumor samples when compared with in adjacent normal tissues (Figure 1C,D). Importantly, HBV infection was associated with higher levels of CCL22 and Foxp3 than no HBV infection (Figure 1C,D), indicating more Tregs (Foxp3 positive) were recruited into the tumor tissues of HBV carriers than noncarriers. The results of CCL22 and Foxp3 protein levels (Figure 1E,F) in each group were consistent with the mRNA results in Figure 1C,D. Next, we examined whether p65, one of the most important transcription regulators in the tumor progression, was involved in the dysregulation of these genes. As shown in Figure 1E,F, p‐p65 and total p65 levels were significantly induced in HBV^+^ and HBV^−^ tumors, and HBV^+^ tumors contained the highest level and normal tissues had lowest level. The pattern of alterations was similar with CCL22 and Foxp3, indicating that p65 might be involved in the regulation of these molecules. Furthermore, the costaining of Foxp3 and CD4 (markers of T cells) were evaluated by immunofluorescent staining in tissues (Figure 1G). The results indicated the CD4^+^Foxp3^+^ cells were significantly increased in both types of tumors (HBV^−^ and HBV^+^) than in normal controls, and HBV infection appeared to more CD4^+^Foxp3^+^ cells. These data implied that the alterations in Foxp3 levels (mRNA and protein) were mostly due to changes in Tregs population in tumor tissues. To explore whether functional interactions existed between these dysregulated molecules, the correlation between miR‐23a and CCL22 or Foxp3 was then computed in three types of tissue samples. It was shown that no significant correlation could be observed between miR‐23a expression and CCL22 or Foxp3 expression in normal group. But, a significant inverse correlation between miR‐23a and CCL22 or Foxp3 was found from HBV^‐^ tumor group and HBV^+^ tumor group (Figure 1H,I).

### 3.2 MiR‐23a, p‐p65, p65, and CCL22 levels were dysregulated in HCC cell lines

**Figure 2:**
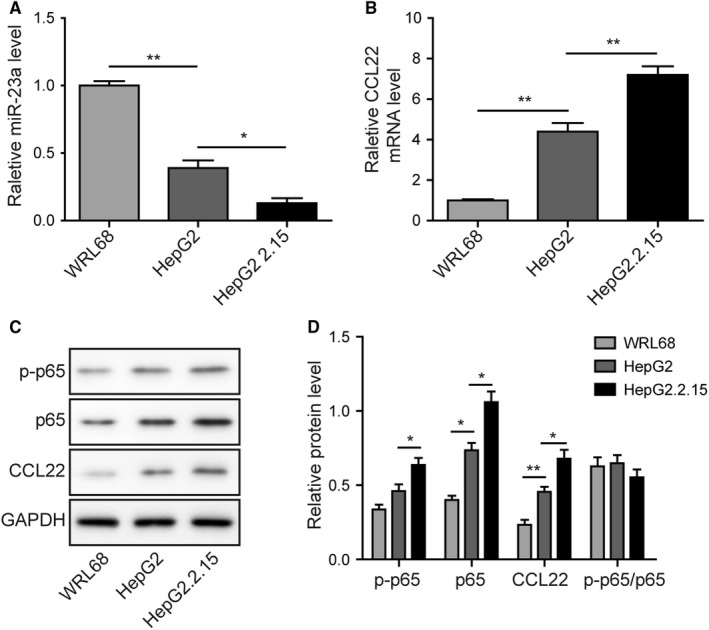
MiR‐23a, p‐p65, p65, and CCL22 levels were dysregulated in HCC cell lines. A, RT‐qPCR revealed that miR‐23a expression was lowest in the HBV‐positive HepG2.2.15 cell line. B, Highest mRNA level of CCL22 was observed in HepG2.2.15. C, Protein levels of p‐p65, p65, and CCL22 in three cell lines were determined by Western blotting. D, The gray scale analysis of p‐p65, p65, CCL22 and ratio of p‐p65/p65 in three cell lines. Expression of CCL22, p‐p65, and p65 were higher in HBV^+^ HepG2.2.15 cells than their parental HBV^‐^ HepG2 cells and normal WRL68 cells. Error bars represented mean ± SD. **P < .01 and *P < .05

Next, the dysregulation of above genes was validated in normal liver cell line and HCC cell lines. In consistent with tissue samples, similar changes in gene expression were also observed among WRL68, HepG2 (HBV^−^) and HepG2.2.15 (HBV^+^) cells. Lower level of miR‐23a was observed in HepG2 and HepG2.2.15 cells than WRL68 cells (Figure 2A), whereas CCL22 mRNA (Figure 2B) and protein levels (Figure 2C,D) were increased in HCC cells. Similarly, both p‐p65 and p65 protein level were upregulated in HepG2 and HepG2.2.15 cells (Figure 2C,D). But, there was no significant difference in the ratio of p‐p65/p65 among three cell lines (Figure 2D). In particular, it was worth to note that HBV infection appeared to be sufficient to downregulate miR‐23a and increase CCL22 and p65 expression, as indicated by the comparison between HepG2.2.15 and their parental cells HepG2. Meanwhile, the results also implied that these cell lines would be good models for the studies of HBV‐mediated tumor progression. HepG2 and HepG2.2.15 were therefore chose for subsequent functional studies.

### 3.3 p65 promoted Tregs recruitment via directly modulating miR‐23a

**Figure 3:**
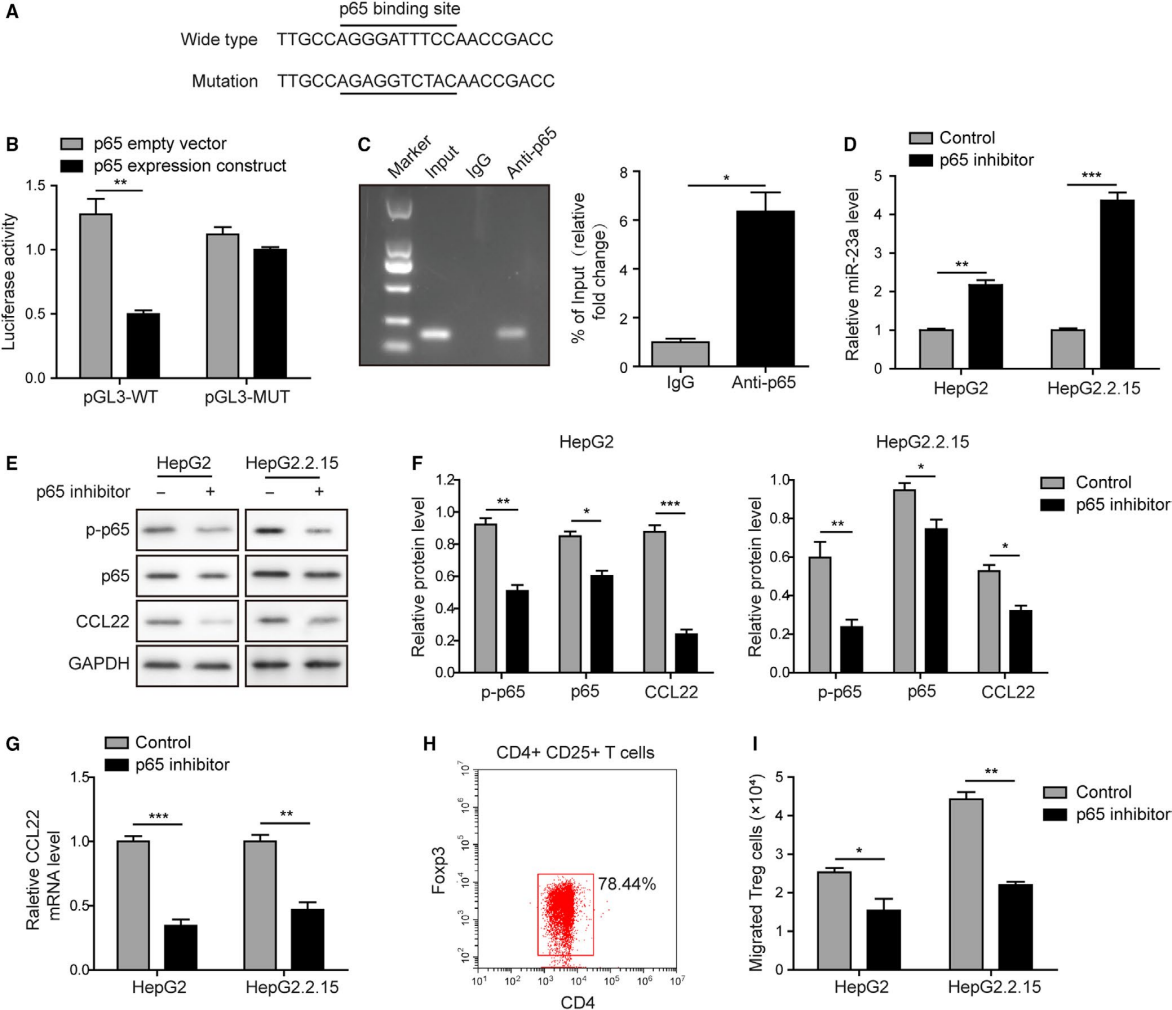
p65 promoted Tregs recruitment via directly modulating miR‐23a. A, A p65‐binding site could be found in miR‐23a promoter region. B, Overexpression of p65 significantly reduced the luciferase activity driven by miR‐23a promoter. Mutagenesis in the p65‐binding site released such repression. C, ChIP assay confirmed that p65 could bind to the promoter region of miR‐23a. D, p65 inhibition upregulated miR‐23a level. E, p65 inhibitor significantly reduced the p‐p65, p65, and CCL22 protein levels in HepG2 and HepG2.2.15 cells. F, The gray scale analysis of p‐p65, p65, CCL22 after p65 inhibitor treatment. G, p65 inhibition dampened CCL22 mRNA level. H, CD4^+^CD25^+^ Foxp3^+^ human Tregs were identified. I, HBV^+^ HepG2.2.15 cells caused more Tregs to migrate through transwell membrane than HBV^−^ HpeG2 cells. Pretreatment with p65 inhibitor attenuated Tregs transmigration in both cell lines. Error bars represented mean ± SD. *P < .05, **P < .01, and ***P < .001

As demonstrated in Figure 1, p65 expression was inversely correlated with miR‐23a expression (Figure 1). To explore the potential interaction of p65 and miR‐23a, we examined whether p65 could act as a transcriptional repressor of miR‐23a in liver cancer. Promoter analysis revealed that a consensus p65‐binding sequence (AGGGATTTCC) was located in the promoter region of miR‐23a. The promoter region containing wide type (WT) or mutated (Mut, AGAGGGTCTAC) p65‐binding site was then subcloned into pGL3‐basic vector (Figure 3A). Dual‐luciferase assay showed that p65‐binding site mutagenesis did not affect the basal activity of promoter (Figure 3B). However, p65 overexpression significantly reduced the wide type of miR‐23a promoter driven luciferase expression, but not its mutant counterpart (Figure 3B). Furthermore, ChIP assay showed that the promoter region of miR‐23a could be significantly enriched by anti‐p65 but not its isotype IgG control (Figure 3C). These results indicated that induction of p65 expression was sufficient to repress miR‐23a promoter activity. Treatment with parthenolide, a p65‐specific inhibitor, disinhibited the expression of miR‐23a (Figure 3D), whereas significantly decreased phosphorylated form of p65 and CCL22 protein and mRNA levels in both tumor cell lines (Figure 3E‐G). Moreover, purified Tregs (Figure 3H) were applied in the transwell migration assay. It should be noted that significantly more Tregs were recruited by HepG2.2.15 cells than HepG2 cells. p65 inhibition by parthenolide treatment greatly attenuated Tregs migration in both HepG2 and HepG2.2.15 cell lines (Figure 3I). We concluded that p65 promoted Tregs recruitment via directly modulating miR‐23a.

### 3.4 MiR‐23a inhibited Tregs recruitment via directly targeting CCL22

**Figure 4:**
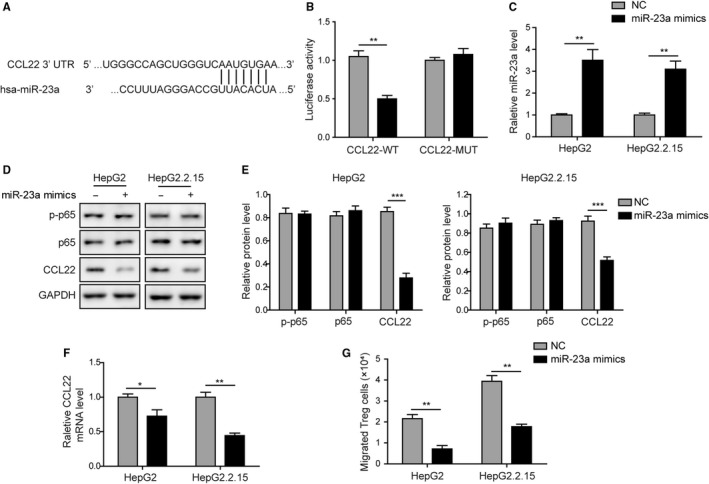
MiR‐23a inhibited Tregs migration by directly targeting CCL22. A, CCL22 3′UTR contains a putative miR‐23a‐binding site. B, MiR‐23a mimics significantly reduced the luciferase activity tagged with wide type of CCL22 3′UTR. Mutagenesis in the miR‐23a‐binding site abolished the inhibitory actions. C, Transfection of miR‐23a mimics efficiently increased miR‐23a level in both cell lines. D, The effects of miR‐23a overexpression on p‐p65, p65, and CCL22 protein levels in HepG2 and HepG2.2.15 cells were assessed by western blotting. E, The gray scale analysis of p‐p65, p65, CCL22 after miR‐23a overexpression. MiR‐23a mimics transfection did not alter the expression of p65 and its phosphorylation but CCL22 was significantly reduced by miR‐23a mimics. F, MiR‐23a mimics significantly reduced CCL22 mRNA level. G, Overexpression of miR‐23a was sufficient to attenuate Tregs transmigration in both cell lines. Error bars represented mean ± SD. *P < .05, **P < .01, and ***P < .001

Next, we studied whether miR‐23a was involved in the CCL22 expression to modulate Tregs recruitment. As mentioned above, CCL22 3′UTR was predicted to contain a 7 bp seed‐binding sequence for miR‐23a (Figure 4A). This region and its mutated counterpart were therefore subcloned into pmirGLO vector and applied for luciferase assay. Cotransfection study revealed that miR‐23a mimics destroyed wide type but not mutated 3′UTR activity of CCL22 (Figure 4B). qPCR assay confirmed that miR‐23a mimics transfection significantly increased miR‐23a level in both cell lines (Figure 4C). While neither p‐p65 nor p65 was altered, miR‐23a mimics did dramatically reduce CCL22 protein and mRNA levels (Figure 4D‐F). As a consequence, miR‐23a mimics also remarkably attenuated Treg cell transmigration (Figure 4G). These results confirmed that CCL22 was a target gene of miR‐23a. It should be noted that neither p65 phosphorylation nor total p65 level responded to miR‐23a mimics. Instead, p65 inhibition could lead to significant upregulation of miR‐23a expression (Figure 3). These results suggested that p65 was an upstream repressor of miR‐23a, which may directly target to CCL22 and further modulate Tregs recruitment.

### 3.5 MiR‐23a inhibitor reversed p65 inhibition effects on CCL22 and Tregs recruitment

**Figure 5:**
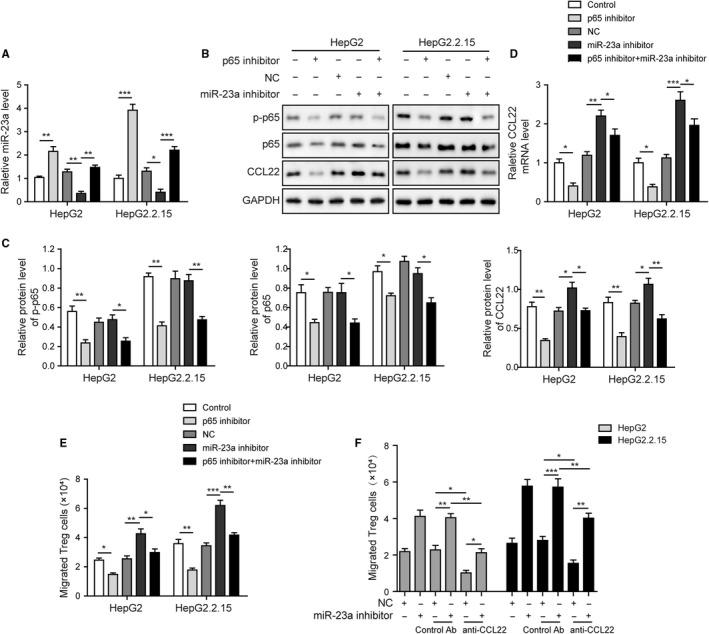
MiR‐23a inhibitor reversed p65 inhibition effects on CCL22 and Tregs recruitment. A, MiR‐23a inhibitor reversed p65 inhibition effects on miR‐23a level. B, The effects of miR‐23a inhibition and p65 inhibitor treatment on p‐p65, p65, and CCL22 protein levels in HepG2 and HepG2.2.15 cells were assessed by western blotting. C, The gray scale analysis of p‐p65, p65, CCL22. MiR‐23a inhibitor reversed p65 inhibition effects on CCL22 protein level but had no effects on p‐p65 and p65. D, MiR‐23a inhibitor reversed p65 inhibition effects on CCL22 mRNA level. E, MiR‐23a inhibitor reversed p65 inhibition effects on Tregs recruitment. F, CCL22 neutralizing antibody reversed miR‐23a inhibitor‐mediated Tregs migration. Error bars represented mean ± SD. *P < .05, **P < .01, and ***P < .001

Given that p65 modulated miR‐23a expression, and that CCL22 was a direct target of miR‐23a, it was speculated that the p65/miR‐23a/CCL22 axis was existed in the liver cancer cells. To this end, cotreatment with p65 inhibitor and miR‐23a inhibitor was applied to the HepG2 and HepG2.2.15 cells. As indicated by the qPCR results, parthenolide significantly upregulated miR‐23a level compared to vehicle control, whereas cotreatment with miR‐23a inhibitor efficiently reversed this action (Figure 5A). Western blotting assay showed that parthenolide alone significantly inhibited the protein levels of p‐p65 and p65 and decreased both protein and mRNA levels of CCL22 (Figure 5B‐D). However, pretreatment with miR‐23a inhibitor caused no effects on p‐p65 and p65 but abolished the repressive effects on CCL22 induced by parthenolide (Figure 5B‐D). These results suggested p65 inhibition reduced CCL22 expression through inducing miR‐23a expression. Finally, consistent changes were also observed in the transmigration assay using Treg cells. MiR‐23a inhibitor treatment alone induced Tregs recruitment. While p65 inhibition significantly reduced Tregs recruitment, pretreatment with miR‐23a inhibitor completely abolished such actions (Figure 5E). Moreover, when coadministrated with anti‐CCL22 neutralizing antibody, the promoting effects of miR‐23a inhibitor on Tregs recruitment were significantly diminished (Figure 5F). Our findings provided evidence that p65‐induced CCL22 induction was mediated by transcriptionally repressing miR‐23a, and that CCL22 was the indispensable effector underlying p65/miR‐23a axis and Tregs recruitment. It was deduced that the axis of p65/miR‐23a/CCL22 was presented in the hepatocellular carcinoma cells and might drive the tumor progression by recruiting Tregs, particularly when HBV infection was involved.

### 3.6 MiR‐23a inhibitor reversed p65 inhibition effects on HBV‐positive xenograft tumor growth

**Figure 6:**
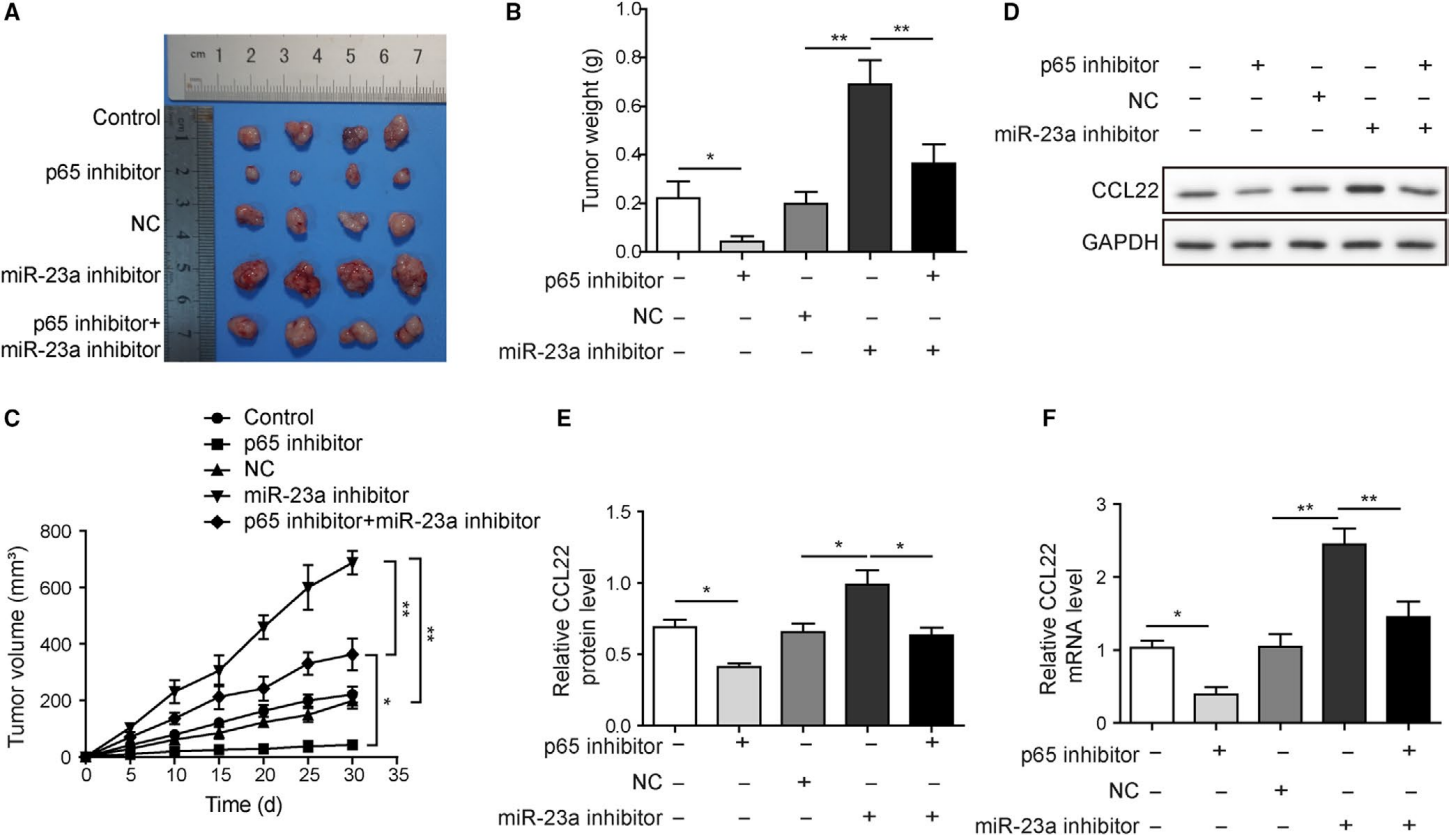
MiR‐23a inhibitor reversed p65 inhibition effects on HBV‐positive xenograft tumor growth. A, Xenografted tumors were harvested after different treatments. B and C, p65 inhibitor treatment significantly retarded tumor growth and miR‐23a inhibitor promoted tumor growth as indicated by the tumor weight and size. The therapeutic effects of p65 inhibitor were abolished by cotreatment with miR‐23a inhibitor. D, CCL22 protein levels from each group were detected by western blotting. E, Statistical results in D. p65 inhibitor led to reduction of CCL22 protein level but miR‐23a inhibitor increased CCL22 level in tumor tissues. F, p65 inhibitor led to reduction of CCL22 mRNA level but miR‐23a inhibitor increased CCL22 mRNA level in tumor tissues. MiR‐23a inhibitor reversed p65 inhibition effects on CCL22 level in tumor tissues. Error bars represented mean ± SD. *P < .05 and **P < .01

The roles of p65/miR‐23a/CCL22 axis was then investigated in the xenograft tumor models. The mice were assigned into five groups according to the treatment. It was shown that p65 inhibitor treatment significantly retarded tumor growth and miR‐23a inhibitor promoted tumor growth as indicated by the tumor weight and size (Figure 6A‐C). In addition, the therapeutic effects induced by p65 inhibitor were dramatically impaired through cotreatment with miR‐23a inhibitor (Figure 6A‐C). This finding coincided with in vitro studies and further strengthened the idea that miR‐23a was the downstream effector of p65 during tumor progression. More importantly, the protein and mRNA levels of CCL22 in the xenograft tumors was highly analogous to the situation of tumor size among groups (Figure 6D‐F). Altogether, we concluded that p65 overactivation could repress miR‐23a expression, leading to CCL22 disinhibition and subsequent promote tumor growth. Targeting the p65/miR‐23a/CCL2 axis represents a novel approach for HBV‐positive HCC therapy.

## 4 DISCUSSION

Around 80% liver cancer patients are HBV carriers in China.^2^ Chronic hepatitis B may develop liver fibrosis, cirrhosis, and HCC eventually.^18^ Although HBV has been identified as a highly tumorigenic factor, the HBV‐featured molecule events for the HCC progression are not clearly understood. One possible reason is that comparison between HBV^‐^ and HBV^+^ liver cancer is lack. In this study, we demonstrated that dysregulation of p65/miR‐23a/CCL22 axis contributed to Tregs recruitment and hepatocellular carcinoma growth. We further showed that HBV infection aggravated the malignancy by enlarging the dysregulation of such axis.

Meta‐analysis revealed that elevation of tumor‐infiltrating Tregs predicted poor prognosis of hepatocellular carcinoma.^6^, ^8^ We found that intratumoral Foxp3 expression was significantly upregulated in HBV^+^ patients in comparison with both healthy control and HBV^−^ patients. Also, the CD4^+^Foxp3^+^ cells were significantly increased in HBV^+^ patients (Figure 1). But, in consideration of the complexity of the tissues, especially in HCC tissues, combined our qPCR and western blotting results in Figure 1C‐F, all these results could suggest that more Tregs were recruited into HBV^+^ tumor tissues. Our observation therefore may highlight intratumoral Tregs as risk factors for poor prognosis and explain the survival analysis which showed that HBV infection caused significant shorter survival time than HBV negative. This was consistent with previous reports showing that Tregs invasion was gradually increased in different stages of tumorigenesis of HCC, from HBV infection, cirrhosis, adenomatous hyperplasia, primary HCC to advance HCC.^19^

Tumor cells‐derived CCL22 was a critical chemoattractant for Tregs recruitment.^8^, ^14^ In this study, we demonstrated that Foxp3 level was associated with the expression of CCL22. Higher Foxp3 in HBV^+^ tumors were accompanied to higher CCL22 level when compared with HBV^‐^ tumors (Figure 1). Consistently, the expression level of CCL22 was higher in HepG2.2.15 cells (HBV^+^) than HepG2 (HBV^−^) and WRL68 (normal) cells. Given that CCL22 acted as an essential attractive signal of Tregs recruitment in various cancers,^14^, ^20^, ^21^ and that Foxp3 was the highly specific marker of Tregs, higher CCL22 and Foxp3 levels in HBV^+^ tumor implied higher intratumoral Tregs infiltration than HBV^‐^ tumors. In contrast, miR‐23a expression was found to be inversely correlated with both CCL22 and Foxp3, particularly in HBV^+^ tumors (Figure 1). Similarly, it was found that miR‐23a level was significantly lower in both types of tumors (HBV‐ and HBV+) than in normal controls (Figure 2). Silico analysis revealed that CCL22 3′UTR contained a binding site of miR‐23a seed sequence. Transfection with miR‐23a mimics dramatically blocked 3′UTR activity. Moreover, miR‐23a mimics efficiently reduced CCL22 expression in both protein and mRNA levels, and attenuated CCL22‐dependent Tregs recruitment (Figure 4). Our study, for the first time, identified miR‐23a as an upstream repressor of CCL22 and Tregs recruitment. Taken together with the functional study and the observation that miR‐23a was significantly lower in tumor tissues than healthy control, we concluded miR‐23a could be a novel tumor suppressor in HCC. This was consistent with the reports that miR‐23a could suppress proliferation of pancreatic cancer cells^22^ and prostate cancer cells.^23^ Notably, differential expression of miR‐23a was also observed between HBV^+^ and HBV^‐^ tumors (Figure 1). The presence of HBV caused even lower miR‐23a expression. Our finding therefore suggested that different clinical outcome between HBV^+^ and HBV^−^ patients was, at least in part, due to the differential miR‐23a reduction.

Despite that a number of microRNAs were shown to hold antitumor potential, the clinical application remains a great challenge. Alternatively, upstream modulators of these microRNAs would be options for the future application. In this study, we demonstrated that p65 was a strong transcriptional repressor of miR‐23a. Inhibition of overactivated p65 could be a promising approach for the recovery of miR‐23a in HCC. In human tissues, phosphorylated and total p65 levels were higher in tumors than normal controls. In cell lines, total p65, and to a less extent, phosphorylated p65 were significantly increased in HepG2 and HepG2.2.15 than WRL68. These results demonstrated that total p65 level was inversely correlated with miR‐23a expression. Dual‐luciferase and ChIP assays revealed that the p65‐binding site in the promoter region of miR‐23a was accessible for p65 (Figure 3). Moreover, Parthenolide, a p65 inhibitor, could reduce CCL22 expression, disinhibit miR‐23a expression and inhibit Tregs migration in vitro (Figure 3). These findings suggested that elevation of p65 was sufficient to repress miR‐23a expression and hence release the brakes on tumorigenesis by miR‐23a. Although phosphorylation has been highlighted to play important roles in the molecular function of p65, it is not always the case. For example, p65 directly bound to the promoter region of miR‐590 and repressed its expression in osteosarcoma, a process independent of p65 phosphorylation.^24^ Alternatively, p65 might act like a scaffold protein and recruit HDAC to miR‐23a promoter, which in turn inhibited the expression of miR‐23a in leukemic Jurkat cells.^25^ We concluded that phosphorylation was not indispensable to p65‐mediated inhibition of miR‐23a expression in HCC. Previously, it has been frequently reported that overactivated p65 signaling pathway was intensively involved in the tumorigenesis of HCC via directly promoting the cell survival and proliferation.^26^, ^27^ Our study therefore proposed a novel mechanism that p65 may facilitate HCC progression via Tregs recruitment.

On the other hand, pretreatment with miR‐23a inhibitor abolished these effects induced by p65 inhibitor, which resulted in reversing the expression of miR‐23a and CCL22 and increasing Tregs recruitment. It also should be noted that Tregs recruitment by miR‐23a inhibition was reversed by CCL22 neutralizing antibody pretreatment (Figure 5). This result strengthened that CCL22 was the downstream effector of p65/miR‐23a axis and Tregs recruitment. Previously, it was demonstrated that upregulated CCL22 was essential for Tregs recruitment and venous metastases of HBV^+^ HCC.^8^ Furthermore, TGF‐β/miR‐34a axis was identified to play a crucial role in the expression of CCL22.^8^ Our study provided a novel mechanism underlying CCL22 modulation by revealing the role of p65/miR‐23a axis. Also, in tumor tissues, HBV infection appeared to cause more dramatical changes in gene expression of p65, miR‐23a and CCL22 than no infection. In particular, the animal study using HepG2.2.15 cells (HBV^+^) suggested that blockage of p65 may efficiently attenuate tumor‐infiltrating Tregs and consequent tumor growth through miR‐23a induction (Figure 6).

## 5 CONCLUSIONS

Our findings provided strong evidences that p65 functioned as an upstream modulator of miR‐23a. Blockage of p65 was sufficient to recover miR‐23a expression in tumor cells, which then repressed CCL22 expression and Tregs recruitment into tumor tissues. To the best of our knowledge, this is the first study to identify the p65/miR‐23a/CCL22 axis as a novel molecular mechanism that could drive intratumoral Tregs recruitment and tumor progression. Our study therefore suggested that targeting p65/miR‐23a/CCL22 axis was a potential approach for the control of HBV^+^ HCC progression.

## CONFLICT OF INTEREST

The authors declare that they have no conflicts of interest.

## Data Availability

All data generated or analyzed during this study are included in this published article.

## References

[1] Chen W , Zheng R , Baade PD , et al. Cancer statistics in China, 2015. CA Cancer J Clin. 2016;66:115‐132. 26808342 10.3322/caac.21338

[2] Yu MC , Yuan JM , Govindarajan S , Ross RK . Epidemiology of hepatocellular carcinoma. Can J Gastroenterol. 2000;14:703‐709. 11185536 10.1155/2000/371801

[3] Spranger S , Gajewski TF . Impact of oncogenic pathways on evasion of antitumour immune responses. Nat Rev Cancer. 2018;18:139‐147. 29326431 10.1038/nrc.2017.117 PMC6685071

[4] Zou W . Immunosuppressive networks in the tumour environment and their therapeutic relevance. Nat Rev Cancer. 2005;5:263‐274. 15776005 10.1038/nrc1586

[5] Zou W . Regulatory T cells, tumour immunity and immunotherapy. Nat Rev Immunol. 2006;6:295‐307. 16557261 10.1038/nri1806

[6] Zhao H‐Q , Li W‐M , Lu Z‐Q , Yao Y‐M . Roles of Tregs in development of hepatocellular carcinoma: a meta‐analysis. World J Gastroenterol. 2014;20:7971‐7978. 24976734 10.3748/wjg.v20.i24.7971 PMC4069325

[7] Shang B , Liu Y , Jiang S , Liu Y . Prognostic value of tumor‐infiltrating FoxP3+ regulatory T cells in cancers: a systematic review and meta‐analysis. Sci Rep. 2015;5:15179. 26462617 10.1038/srep15179 PMC4604472

[8] Yang P , Li Q‐J , Feng Y , et al. TGF‐β‐miR‐34a‐CCL22 signaling‐induced Treg cell recruitment promotes venous metastases of HBV‐positive hepatocellular carcinoma. Cancer Cell. 2012;22:291‐303. 22975373 10.1016/j.ccr.2012.07.023 PMC3443566

[9] Chen L , Zhou S , Qin J , et al. Combination of SLC administration and Tregs depletion is an attractive strategy for targeting hepatocellular carcinoma. Mol Cancer. 2013;12:153. 24304581 10.1186/1476-4598-12-153 PMC3914677

[10] Makarova‐Rusher OV , Medina‐Echeverz J , Duffy AG , Greten TF . The yin and yang of evasion and immune activation in HCC. J Hepatol. 2015;62:1420‐1429. 25733155 10.1016/j.jhep.2015.02.038

[11] Farkona S , Diamandis EP , Blasutig IM . Cancer immunotherapy: the beginning of the end of cancer? BMC Med. 2016;14:73. 27151159 10.1186/s12916-016-0623-5 PMC4858828

[12] Yoshie O , Matsushima K . CCR4 and its ligands: from bench to bedside. Int Immunol. 2015;27:11‐20. 25087232 10.1093/intimm/dxu079

[13] Martinenaite E , Munir Ahmad S , Hansen M , et al. CCL22‐specific T Cells: Modulating the immunosuppressive tumor microenvironment. Oncoimmunology. 2016;5:e1238541. 27999757 10.1080/2162402X.2016.1238541 PMC5139648

[14] Gobert M , Treilleux I , Bendriss‐Vermare N , et al. Regulatory T cells recruited through CCL22/CCR4 are selectively activated in lymphoid infiltrates surrounding primary breast tumors and lead to an adverse clinical outcome. Cancer Res. 2009;69:2000‐2009. 19244125 10.1158/0008-5472.CAN-08-2360

[15] Anz D , Rapp M , Eiber S , et al. Suppression of intratumoral CCL22 by type I interferon inhibits migration of regulatory T cells and blocks cancer progression. Cancer Res. 2015;75:4483‐4493. 26432403 10.1158/0008-5472.CAN-14-3499

[16] Wang N , Zhu M , Wang X , Tan H‐Y , Tsao S , Feng Y . Berberine‐induced tumor suppressor p53 up‐regulation gets involved in the regulatory network of MIR‐23a in hepatocellular carcinoma. Biochim Biophys Acta ‐ Gene Regul Mech. 1839;2014:849‐857. 10.1016/j.bbagrm.2014.05.027 24942805

[17] Guo W , Wang H , Yang Y , et al. Down‐regulated miR‐23a contributes to the metastasis of cutaneous melanoma by promoting autophagy. Theranostics. 2017;7:2231‐2249. 28740547 10.7150/thno.18835 PMC5505056

[18] Sundaram V , Kowdley K . Management of chronic hepatitis B infection. BMJ. 2015;351:h4263. 26491030 10.1136/bmj.h4263

[19] Kobayashi N , Hiraoka N , Yamagami W , et al. FOXP3+ regulatory T cells affect the development and progression of hepatocarcinogenesis. Clin Cancer Res. 2007;13:902‐911. 17289884 10.1158/1078-0432.CCR-06-2363

[20] Klarquist J , Tobin K , Oskuei PF , et al. Microenvironment and immunology Ccl22 diverts T regulatory cells and controls the growth of Melanoma. Cancer Res. 2016;76(21):6230‐6240. 27634754 10.1158/0008-5472.CAN-16-0618 PMC5242486

[21] Li Y‐Q , Liu F‐F , Zhang X‐M , Guo X‐J , Ren M‐J , Fu L . Tumor secretion of CCL22 activates intratumoral Treg infiltration and is independent prognostic predictor of breast cancer. PLoS ONE. 2013;8:e76379. 24124553 10.1371/journal.pone.0076379 PMC3790712

[22] Chen B , Zhu A , Tian L , et al. miR‐23a suppresses pancreatic cancer cell progression by inhibiting PLK‐1 expression. Mol Med Rep. 2018;18:105‐112. 29749476 10.3892/mmr.2018.8941 PMC6059658

[23] Aghaee‐Bakhtiari SH , Arefian E , Naderi M , et al. MAPK and JAK/STAT pathways targeted by miR‐23a and miR‐23b in prostate cancer: computational and in vitro approaches. Tumor Biol. 2015;36:4203‐4212. 10.1007/s13277-015-3057-3 25604141

[24] Long X , Lin X‐J . P65‐mediated miR‐590 inhibition modulates the chemoresistance of osteosarcoma to doxorubicin through targeting wild‐type p53‐induced phosphatase 1. J Cell Biochem. 2019;120:5652‐5665. 30387173 10.1002/jcb.27849

[25] Rathore MG , Saumet A , Rossi J‐F , et al. The NF‐κB member p65 controls glutamine metabolism through miR‐23a. Int J Biochem Cell Biol. 2012;44:1448‐1456. 22634383 10.1016/j.biocel.2012.05.011

[26] He G , Karin M . NF‐κB and STAT3 ‐ key players in liver inflammation and cancer. Cell Res. 2011;21:159‐168. 21187858 10.1038/cr.2010.183 PMC3193410

[27] Luedde T , Schwabe RF . NF‐κB in the liver–linking injury, fibrosis and hepatocellular carcinoma. Nat Rev Gastroenterol Hepatol. 2011;8:108‐118. 21293511 10.1038/nrgastro.2010.213 PMC3295539

